# Potentiating Hemorrhage in a Periadolescent Rat Model of Closed-Head Traumatic Brain Injury Worsens Hyperexcitability but Not Behavioral Deficits

**DOI:** 10.3390/ijms22126456

**Published:** 2021-06-16

**Authors:** Dounya Jalloul, Helene Hajjar, Rita Asdikian, Mariam Maawie, Leila Nasrallah, Yasser Medlej, Mouhamad Darwich, Nabil Karnib, Nada Lawand, Ronza Abdel Rassoul, Kevin K. W. Wang, Firas Kobeissy, Hala Darwish, Makram Obeid

**Affiliations:** 1 Department of Anatomy, Cell Biology and Physiological Sciences, American University of Beirut, Beirut 1107 2020, Lebanon; dfj04@mail.aub.edu (D.J.); helenehajjar@outlook.com (H.H.); rha69@mail.aub.edu (R.A.); yasser.medlej@uks.eu (Y.M.); nkarnib0@gmail.com (N.K.); n108@aub.edu.lb (N.L.); 2 Neuroscience Research Center, Faculty of Medical Sciences, Lebanese University, Hadath P.O. Box 6573/14, Lebanon; mariam_ma96@hotmail.com (M.M.); ronza.abdelrassoul@gmail.com (R.A.R.); 3 Department of Biochemistry and Molecular Genetics, American University of Beirut, Beirut 1107 2020, Lebanon; ln23@aub.edu.lb; 4 Division of Child Neurology, Department of Pediatrics and Adolescent Medicine, American University of Beirut Medical Center, Beirut 1107 2020, Lebanon; mouhamad.el.darwich@gmail.com; 5 Department of Neurology, American University of Beirut Medical Center, Beirut 1107 2020, Lebanon; 6 Brain Rehabilitation Research Center, Malcom Randall VA Medical Center, Gainesville, FL 32608, USA; kawangwang17@gmail.com; 7 Program for Neurotrauma, Neuroproteomics & Biomarkers Research, Departments of Emergency Medicine, University of Florida, Gainesville, FL 32608, USA; 8 Rafic Hariri School of Nursing, American University of Beirut, Beirut 1107 2020, Lebanon

**Keywords:** controlled cortical impact, hemorrhage, hyperexcitable, astrocyte, adolescent, spike, behavior, epilepsy

## Abstract

Post-traumatic epilepsy (PTE) and neurocognitive deficits are devastating sequelae of head injuries that are common in adolescents. Investigating desperately needed treatments is hindered by the difficulties in inducing PTE in rodents and the lack of established immature rat models of pediatric PTE. Hemorrhage is a significant risk factor for PTE, but compared to humans, rats are less prone to bleeding because of their rapid blood coagulation system. In this study, we promoted bleeding in the controlled cortical impact (CCI) closed-head injury model with a 20 min pre-impact 600 IU/kg intraperitoneal heparin injection in postnatal day 35 (P35) periadolescent rats, given the preponderance of such injuries in this age group. Temporo-parietal CCI was performed post-heparin (HTBI group) or post-saline (TBI group). Controls were subjected to sham procedures following heparin or saline administration. Continuous long-term EEG monitoring was performed for 3 months post-CCI. Sensorimotor testing, the Morris water maze, and a modified active avoidance test were conducted between P80 and P100. Glial fibrillary acidic protein (GFAP) levels and neuronal damage were also assessed. Compared to TBI rats, HTBI rats had persistently higher EEG spiking and increased hippocampal GFAP levels (*p* < 0.05). No sensorimotor deficits were detected in any group. Compared to controls, both HTBI and TBI groups had a long-term hippocampal neuronal loss (*p* < 0.05), as well as contextual and visuospatial learning deficits (*p* < 0.05). The hippocampal astrogliosis and EEG spiking detected in all rats subjected to our hemorrhage-promoting procedure suggest the emergence of hyperexcitable networks and pave the way to a periadolescent PTE rat model.

## 1. Introduction

Traumatic brain injury (TBI) is common in adolescents and adults, with 300–400 cases per 100,000 individuals [1]. TBI is associated with detrimental neurological sequelae, including post-traumatic epilepsy (PTE), as well as behavioral and cognitive deficits [2,3]. The incidence of PTE is as high as 53% following severe head injuries [4], and it accounts for up to 20% of all symptomatic epilepsies [5]. The adolescent population is one of the most affected age groups by TBI, and specifically non-penetrating closed-head injuries [6]. Moreover, PTE in the pediatric age group is often associated with severe neurocognitive disorders irrespective of injury severity [7,8]. In current clinical practice, anti-seizure medications are transiently administered for one week following TBI to prevent early post-traumatic seizures in the first 7 days post-injury [9], but to date, there are no available neuroprotective treatment strategies that can prevent the later emergence of PTE and the often accompanying neurocognitive comorbidities. Investigating desperately needed novel preventive treatment strategies is hindered by the lack of well-established pediatric PTE models [10] and the very low rates of PTE emergence in current immature and adult animal models, with as little as 3–9% of experimental animals developing seizures [10,11,12].

Hemorrhage constitutes a significant risk factor for PTE [13,14]. Indeed, up to 84% of PTE patients have prominent hemorrhagic changes on brain imaging [15]. Inducing hemorrhage in TBI animal models is challenging because rodents, the most commonly used animals in TBI and seizure models, are resistant to hemorrhage. Indeed, the rat’s clotting time is fourfold shorter than in humans [16]. Due to the challenges in inducing epilepsy in TBI animal models, we introduced novel modifications to the closed-head cortical impact (CCI) model to promote hemorrhage, hyperexcitability, and PTE emergence in periadolescent postnatal day 35 (P35) rats, given the preponderance of closed-head injuries in this age group. We therefore investigated the effects of a single 20 min pre-impact injection of the anticoagulant heparin, on potential CCI-induced epileptiform features, seizures, behavioral deficits [6], neuronal loss, as well as reactive astrocytosis, given its implication in hyperexcitability and epileptogenesis [17,18,19,20,21].

## 2. Results

### 2.1. CCI with Prominent Hemorrhage

**Figure 1:**
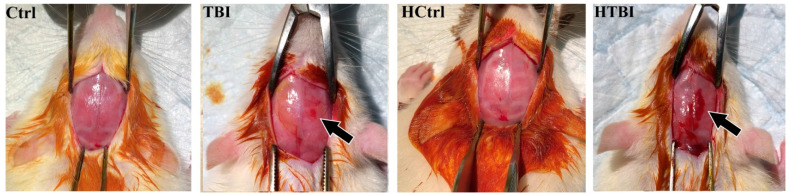
Post-impact hemorrhage. Shown are images of the exposed skull obtained immediately following controlled cortical impact (CCI) with black arrows pointing at the traumatic impact site in the HTBI (traumatic brain injury post-heparin) and TBI (traumatic brain injury post-saline) groups. Following our modified CCI post-heparin procedure, all rats in the HTBI group developed visibly prominent fresh blood under the skull at the impact site (arrow), without detectable ongoing gross active bleeding. Bruising with minimal hemorrhage at the impact site was seen in the TBI group. No hemorrhage was seen in controls (Ctrl) and heparinized controls (HCtrl).

Visual inspection of the skull immediately following the traumatic impact revealed that our modified CCI post-heparin procedure markedly potentiated impact-associated hemorrhage, as evidenced by visibly prominent fresh blood under the skull at the impact site in all rats in the HTBI group (traumatic brain injury post-heparin, *n* = 16), as shown in Figure 1. On the other hand, there was only bruising at the impact site in the TBI group (traumatic brain injury post-saline, *n* = 15), following the standard CCI post-saline procedure. No hemorrhage was observed in the rats subjected to sham procedures in the HCtrl (heparinized controls, *n* = 16), and the Ctrl (non-heparinized controls, *n* = 12) groups following heparin or normal saline injections, respectively. All rats survived the traumatic impact with no skull fractures or visible indentations.

### 2.2. EEG Features

**Figure 2:**
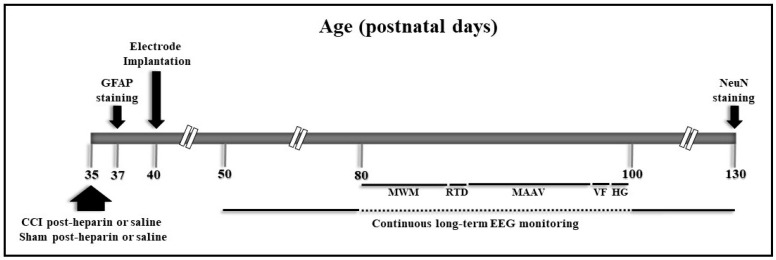
Experimental design. Postnatal day 35 (P35) rats were subjected to closed-head controlled cortical impact (CCI) 20 min post-heparin or post-saline injections. Controls were sham treated post-heparin or post-saline injections. Following epidural electrode implantation surgery at P40, rats underwent long-term continuous EEG monitoring from P50 to P80 and from P100 to P130. The EEG recording was interrupted between P80 and P100 to conduct behavioral testing. The behavioral testing panel consisted of the Morris water maze (MWM) at P80-85, the rotarod (RTD) motor test at P86, the modified active avoidance (MAAV) at P88-94, the Von Frey (VF), and the Hargreaves (HG) sensory tests at P96 and P97, respectively. At P130, rats were sacrificed to assess for neuronal loss with neuronal nuclei (NeuN) staining. A subgroup of rats was sacrificed 48 h post-CCI for the assessment of reactive astrogliosis with glial fibrillary acidic protein (GFAP) staining.

**Figure 3:**
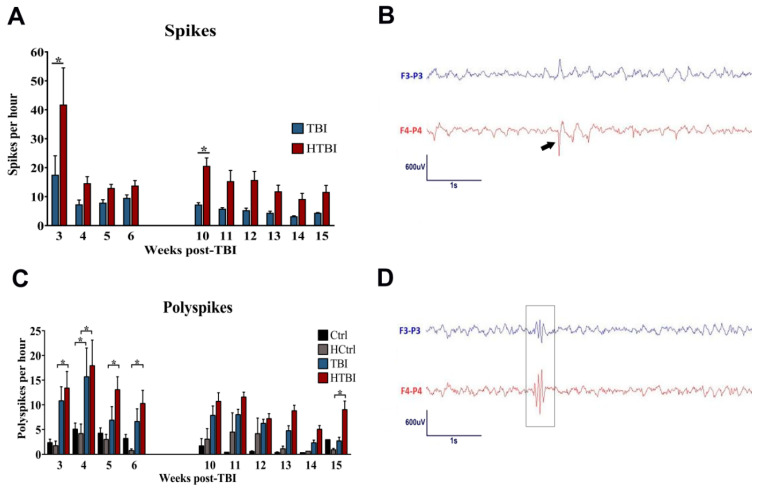
Analyses of the continuous long-term electroencephalogram (EEG) tracings. Spikes and polyspikes were quantified in randomly sampled two-hour epochs per day, one hour from the daytime and one hour from the nighttime. The EEG tracing was interrupted between the 6th and 10th week post-injury to perform behavioral studies: (**A**) spikes were detected in both injured groups, with a higher frequency of spikes in the HTBI group compared to the TBI group, with statistical significance at 3 and 10 weeks post-TBI (*p* < 0.05), as revealed by two-way ANOVA with post hoc Fisher’s least significant difference (LSD). The frequency of spikes dropped in the 4th week post-TBI and plateaued thereafter. No spikes were detected in control rats; (**B**) shown is the EEG tracing of one rat with four sampling electrodes (F3: left frontal, F4: right frontal, P3: left parietal, P4: right parietal), in the longitudinal bipolar montage. The arrow points to a spike morphologically characterized by a pointed peak, followed by a repolarization wave. The spike has a higher amplitude over, and therefore localizes to, the right hemisphere, which is the side of the traumatic impact; (**C**) polyspikes were detected in all groups. The HTBI group had a significantly higher polyspike frequency, compared to its control, the HCtrl group, in the first month of EEG recording, as well as in the EEG tracing obtained in the last week of the study (*p* < 0.05, two-way ANOVA with post hoc Fisher’s LSD test). The polyspike frequency in the TBI group was statistically comparable (*p* > 0.05) to its control (Ctrl) except in the tracing obtained in the 4th week of the recording (*p* < 0.05, two-way ANOVA with Fisher’s LSD test). The frequency of polyspikes peaked in the 4th week post-injury (15.7 ± 5.7 spikes/h for TBI, and 18 ± 5.1 spikes/hr for HTBI), following which it plateaued after the 6th week of recording in the HTBI group but continued to drop gradually in the TBI group; (**D**) shown is the EEG tracing of one rat with four sampling electrodes (F3: left frontal, F4: right frontal, P3: left parietal, P4: right parietal), in the longitudinal bipolar montage. The arrow points to a polyspike. Polyspikes consist of two or more spike components. Asterisks indicate statistically significant differences (*p* < 0.05). Mean ± SEM are represented. (Ctrl: control, *n* = 5; HCtrl: heparinized control, *n* = 5; TBI: traumatic brain injury post-saline, *n* = 9; HTBI: traumatic brain injury post-heparin, *n* = 11).

Long-term continuous electroencephalography (EEG) monitoring was conducted from P50 to P130, with an interruption between P80 and P100 to conduct behavioral studies, as delineated in the schematic study design (Figure 2). A review of the entire EEG tracings did not reveal seizures in any of the groups, but spikes were detected in all the rats in the TBI and HTBI groups. No spikes were detected in any of the Ctrl or HCtrl rats (Figure 3A). Spikes were morphologically distinguishable from background activity, consisted of sharply contoured waves (duration < 80 ms) of relatively high amplitude compared to the baseline, and were followed by repolarization waves. These electrical discharges had a higher amplitude in, and thus localized to, the ipsilateral side of the injury (right side) (Figure 3B). Quantification of the sampled EEG segments (2 h per day) revealed a higher number of spikes in the HTBI rats compared to the TBI group throughout the recording, with a statistically significant difference between these two groups during the first and the tenth week of recording (*p* < 0.05), as revealed by two-way analysis of variance (ANOVA).

Polyspikes were detected in all groups (Figure 3C). These discharges consisted of polyphasic spikes with two or more spike components that diffusely co-occurred in both hemispheres but were at times right predominant in the rats subjected to CCI (Figure 3D). The HTBI group had a significantly higher frequency of polyspikes, compared to its control, HCtrl, in the entire first 4 weeks post-injury, and this significantly higher polyspike frequency was persistent on the last week of the recording (*p* < 0.05, two-way ANOVA). On the other hand, compared to its control (Ctrl) group, the TBI group only had a higher polyspike frequency in the tracing obtained in the 4th week post-injury (*p* < 0.05, two-way ANOVA), and an otherwise statistically comparable polyspike frequency compared to the Ctrl group throughout the remaining EEG tracings.

### 2.3. Behavioral Testing

**Figure 4:**
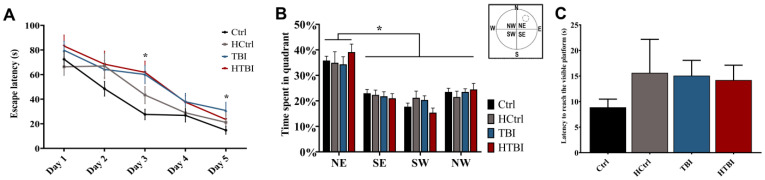
Learning deficits in the Morris water maze (MWM) test: (**A**) shown is the latency to reach the escape platform during the five training days for place learning. Both the HTBI and TBI groups were slower in reaching their full potential on the last training days compared to their respective controls, the HCtrl and Ctrl. Two-way repeated-measures ANOVA with post hoc Fisher’s least significant difference (LSD) revealed that the TBI group was significantly slower than its control group (Ctrl) in reaching the escape platform on days 3 and 5. The HTBI and the TBI groups had a similar performance in learning the place of the platform on all five testing days, as measured by statistically comparable average escape latencies (*p* > 0.05, two-way repeated-measures ANOVA with post hoc Fisher’s LSD); (**B**) in the probe trial test, all groups showed comparable retention of spatial learning by spending significantly more time in the quadrant, where the escape platform was previously located (*p* < 0.05, one-way ANOVA with post hoc Fisher’s LSD). The dashed circle in the water maze diagram corresponds to the previous location of the platform (N: north, E: east, S: south, W: west, NE: northeast, NW: northwest, SE: southeast, SW: southwest); (**C**) all rats had comparable latencies in reaching the visible platform indicating comparable visual and motor functions in all groups (*p* > 0.05, one-way ANOVA with post hoc Fisher’s LSD). Asterisks indicate statistically significant differences (*p* < 0.05). Mean ± SEM are represented. (Ctrl: control, *n* = 12; HCtrl: heparinized control, *n* = 16; TBI: traumatic brain injury post-saline, *n* = 15; HTBI: traumatic brain injury post-heparin, *n* = 16).

Visuospatial learning was assessed in the Morris water maze (MWM). All rats gradually learned to reach the escape platform over the five training days (Figure 4). The escape latencies of both HTBI and TBI were statistically comparable (*p* > 0.05), and significantly slower than controls on days 3 and 5 (*p* < 0.05, two-way ANOVA). In the probe trial test, all groups showed comparable spatial memory retention (*p* > 0.05, one-way ANOVA). The escape latencies to the visible platform were comparable in all groups (*p* > 0.05, one-way ANOVA), indicating no significant visual or locomotor deficits.

**Figure 5:**
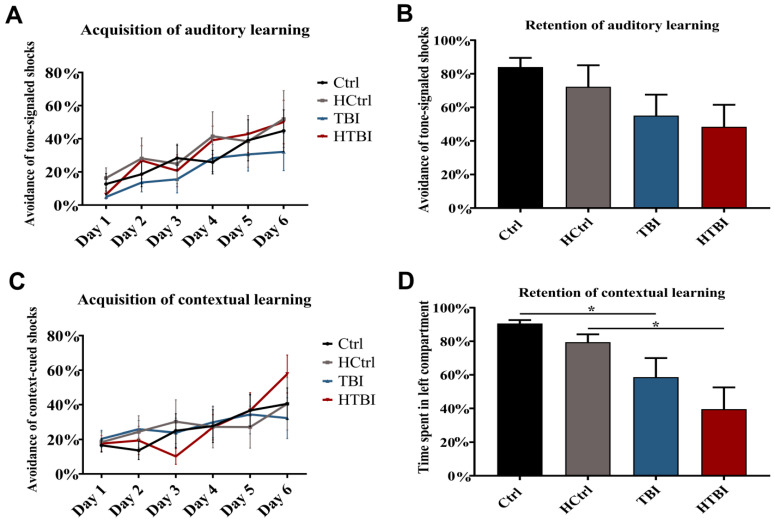
Learning deficits in the modified active avoidance (MAAV) test. Repeated measures two-way ANOVA with post hoc Fisher’s least significant difference (LSD) revealed that all groups were comparable in learning to avoid the tone-signaled electrical foot-shocks in the left chamber (*p* > 0.05) (**A**). There was a trend for both TBI and HTBI groups to have lower retention of auditory learning, compared to their respective controls, but there was not a statistically significant difference (**B**). In the acquisition of contextual learning subtest, all groups were comparable in learning to avoid the context-cued electrical foot shocks (**C**) in the right chamber (*p* > 0.05, two-way ANOVA with repeated measures with post hoc Fisher’s LSD). However, both TBI and HTBI groups had deficits in the retention of contextual learning (**D**), as revealed by statistically significant lower times spent on the left side, compared to their respective controls (90.62 ± 2.00% for Ctrl, 79.47 ± 4.66% for HCtrl, 58.65 ± 11.33% for TBI, 39.59 ± 12.94% for HTBI, *p* < 0.05, one-way repeated measures ANOVA with post hoc Fisher’s LSD). There were no statistically significant differences in all other paired comparisons. Asterisks indicate statistically significant differences (*p* < 0.05). Mean ± SEM are represented. (Ctrl: control, *n* = 12; HCtrl: heparinized control, *n* = 16; TBI: traumatic brain injury post-saline, *n* = 15; HTBI: traumatic brain injury post-heparin, *n* = 16).

Learning of adaptive avoidance behaviors of tone-signaled or context-cured shocks was investigated in the modified active avoidance (MAAV) test (Figure 5). All groups were statistically comparable in learning (Figure 5A) and retaining (Figure 5B) to avoid the tone-signaled electrical foot shocks (*p* > 0.05, two-way ANOVA), even though there was a trend for both TBI and HTBI groups to have lower auditory retention rates. All the rats also learned to avoid the context-cued electrical foot shocks in the right chamber (*p* > 0.05, two-way ANOVA) (Figure 5C); however, both HTBI and TBI groups had statistically significant deficits in the retention of contextual learning (Figure 5D), compared to their respective controls, HCtrl and Ctrl (*p* < 0.05, one-way ANOVA).

Given the parietotemporal location of the impact over the sensory area, and its adjacency to the motor area, motor (rotarod) and sensory (Von Frey and Hargreaves tests) testing were also performed following the MWM and MAAV, respectively, to assess for potential deficits that may affect the swimming performance in the MWM or the sensory abilities in the MAAV. All groups had comparable latencies to fall from the accelerating rotating rod (*p* > 0.05, one-way ANOVA), suggesting comparable motor capabilities (Supplementary Figure S1). In the Von Frey filaments test (Supplementary Figure S2A), all rats had a comparable response rate to the mechanical stimuli in both hind paws (*p* > 0.05, two-way ANOVA). In the Hargreaves test (Supplementary Figure S2B), all animals had comparable withdrawal latencies from thermal stimuli (*p* > 0.05, two-way ANOVA). 

### 2.4. Histological Analyses

**Figure 6:**
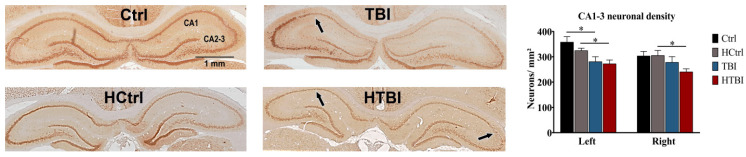
Assessment of neuronal densities. Shown are neuronal nuclei (NeuN) stained histological coronal hippocampal sections. Neuronal densities were calculated by dividing the number of NeuN positive cells into the CA1-3 regions over the hippocampal surface area. Analyses were conducted on 3–5 animals from each group and 6 sections were obtained from each brain. The HTBI and TBI groups had comparable left and right CA1-3 hippocampal neuronal densities (*p* > 0.05), as revealed by one-way ANOVA with post hoc Fisher’s least significance difference (LSD). In the left hippocampus, both HTBI and TBI rats had a significantly lower neuronal density, compared to their respective controls, HCtrl and Ctrl (*p* < 0.05, one-way ANOVA with post hoc Fisher’s LSD). In the right hippocampus, the HTBI group had significantly lower CA1-3 neuronal densities (*p* < 0.05) compared to its control, the HCtrl group, but the TBI group was comparable to its control (*p* > 0.05) on that side (one-way ANOVA with post hoc Fisher’s LSD). The arrows are pointing to the areas of most prominent neuronal loss and thinning in the CA1-3 regions. Analyses of the cortical areas revealed that the right and left parietal cortical neuronal densities were minimally but significantly lower in the HTBI group (604.2 ± 8.3 neurons/mm^2^, and 606.4 ± 8.1 neurons/mm^2^, respectively), compared to all other groups (642.4 ± 16.1 neurons/mm^2^ on the right and 669.1 ± 14.5 neurons/mm^2^ on the left for TBI; 656.4 ± 15.4 neurons/mm^2^ on the right and 647.1 ± 9.3 neurons/mm^2^ on the left for Ctrl; 634.3 ± 11.1 neurons/mm^2^ on the right and 652.4 ± 11.9 neurons/mm^2^ on the left for HCtrl, *p* < 0.05, one-way ANOVA). The TBI group had comparable bilateral parietal cortical neuronal densities to control rats (*p* > 0.05, one-way ANOVA). Asterisks indicate statistically significant differences (*p* < 0.05). Mean ± SEM are represented. (Ctrl: control; HCtrl: heparinized control; TBI: traumatic brain injury post-saline; HTBI: traumatic brain injury post-heparin).

Compared to their respective controls, rats in both HTBI and TBI groups had significantly lower left hippocampal pyramidal neuronal densities (*p* < 0.05, one-way ANOVA). The HTBI group also had a significantly lower right hippocampal neuronal density, compared to its control (*p* < 0.05, one-way ANOVA). Analyses of the cortical areas revealed that the parietal cortical neuronal densities were minimally but significantly lower in the HTBI group bilaterally, compared to all other groups (*p* < 0.05, one-way ANOVA) (Figure 6).

**Figure 7:**
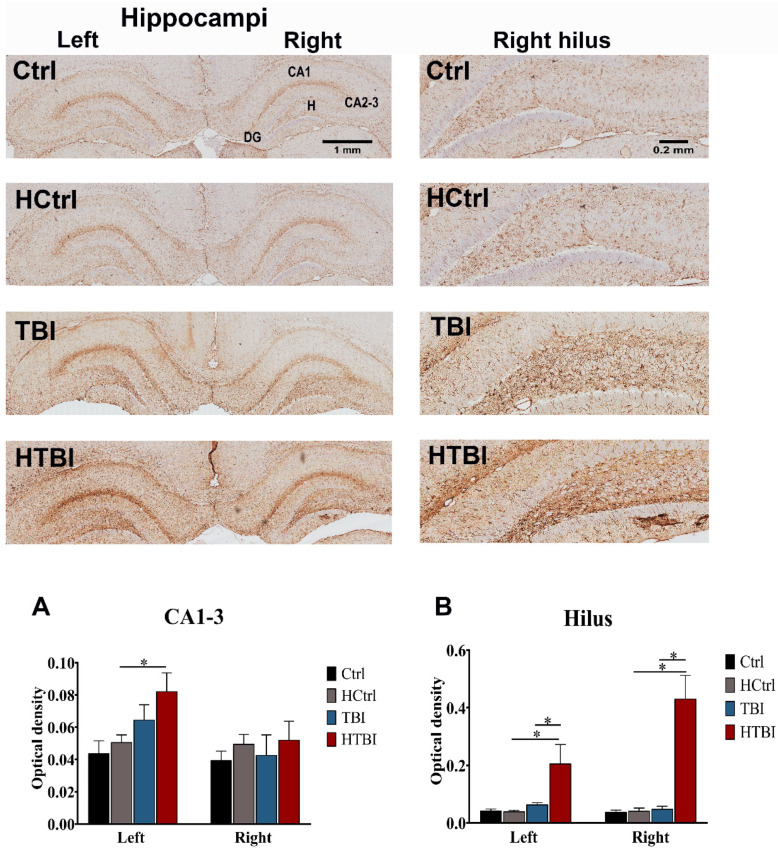
Immunohistochemical assessment of reactive astrogliosis with GFAP (glial fibrillary acidic protein) levels. Shown are illustrative images of coronal hippocampal sections as well as high-power images of the right hippocampal hilar zone ipsilaterally to the impact site. Higher levels of darkly stained astrocytes were observed in the hippocampi of TBI and HTBI rats and more so in the hilar regions bilaterally. In order to quantify this effect, we analyzed the levels of GFAP with optical density measurements on four sections from each brain (*n* = 3–5 rats per group). One-way ANOVA with post hoc Fisher’s least significant difference (LSD) revealed a statistically significant increase in CA1-3 optical density in the HTBI group, compared to its control, HCtrl (*p* < 0.05), on the left but not the right side (**A**), with no other statistically significant differences in paired comparisons between TBI and HTBI groups or between TBI and its control. Analyses of the hilar regions (**B**) revealed statistically significant higher optical densities in the HTBI group, compared to the TBI group and controls bilaterally (*p* < 0.05), while the TBI group had comparable hilar optical densities to controls (*p* > 0.05, one-way ANOVA with post hoc Fisher’s LSD). Analyses of the cortical areas revealed no statistically significant differences between groups (left cortex: 0.059 ± 0.002 for Ctrl, 0.072 ± 0.009 for HCtrl, 0.080 ± 0.016 for TBI, 0.066 ± 0.007 for HTBI; right cortex: 0.063 ± 0.003 for Ctrl, 0.063 ± 0.007 for HCtrl, 0.083 ± 0.012 for TBI, 0.066 ± 0.009 for HTBI, *p* > 0.05, one-way ANOVA with post hoc Fisher’s LSD). Asterisks indicate statistically significant differences (*p* < 0.05). Mean ± SEM are represented. (Ctrl: control; D: dentate gyrus, H: hilus; HCtrl: heparinized control; TBI: traumatic brain injury post-saline; HTBI: traumatic brain injury post-heparin).

As reactive astrogliosis to injury is associated with increased epileptogenicity, we measured cortical and hippocampal glial fibrillary acidic protein (GFAP) levels 48 h post-CCI (Figure 7). In both the left and right hippocampal hilar regions, the HTBI group had significantly increased astrogliosis, compared to all other groups (*p* < 0.05, one-way ANOVA). In the CA1-3 hippocampal areas, there was only a statistically significant increase in GFAP levels on the left side in the HTBI group when compared to control groups (*p* < 0.05, one-way ANOVA). All groups had statistically comparable GFAP levels in the cortex (*p* > 0.05, one-way ANOVA). 

## 3. Discussion

In this study, we modified the traditional CCI model to induce a closed-head injury and increase TBI-associated hemorrhage with a single pre-impact heparin injection, which resulted in the potentiation of post-injury hyperexcitability. Compared to standard CCI, rats subjected to our novel modified CCI procedure had a more prominent impact-associated hemorrhage, and significant increases in EEG and histological markers of hyperexcitability and emerging epileptic networks, namely, increased EEG spiking and reactive hippocampal astrogliosis. Our modified procedure also resulted in increased cortical damage but did not worsen the CCI-associated behavioral deficits. These novel findings echo the clinically described role of hemorrhage in significantly accentuating hyperexcitability in TBI patients [13,14,15] and pave the way for overcoming the challenge of modeling PTE in periadolescent rodents following closed-head injury. 

The promotion of hemorrhage in our novel modified CCI procedure was associated with persistent and significantly increased EEG discharges in the HTBI rats, as shown in Figure 3, suggesting the emergence of a potentially permanent abnormal network of hyperexcitability. Given that not all discharges detectable on EEG are epileptiform in nature and necessarily indicate a brain insult-related emergence of an epileptogenic network [12,22], we morphologically stratified the discharges detected in our modified CCI model into spikes and polyspikes, and independently interpreted them. Since polyspikes were detected in all groups, including controls, they likely represent baseline pre-existent diffuse excitability features that were accentuated by trauma, and more prominently so with the promotion of hemorrhage in the HTBI rats. While epileptiform features that synchronously occur in both hemispheres of control rats in a similar manner to the herein-described polyspikes [12] may represent an inherent genetic epileptogenicity, more research is required to fully elucidate their nature. Unlike polyspikes, spikes were exclusively detected post-CCI, in TBI and HTBI groups but not in controls, and therefore, they reflect an acquired brain-insult-related hyperexcitable circuitry, especially that they localized to the hemisphere subjected to the impact. Compared to standard CCI in the TBI group, the promotion of hemorrhage significantly increased the frequency of spikes in HTBI rats. This high rate of EEG spiking persisted throughout the 3 months of recording and occurred in all the rats subjected to our modified CCI procedure, as opposed to around 85% of animals following CCI in prior studies on adult and immature rodents [23]. In human subjects, EEG spikes are used in supporting the diagnosis of epilepsy, localizing epileptic foci, and guiding therapy in standard clinical practice [24,25,26], but recent research data point to their role in predicting the emergence of PTE following TBI [15]. In preclinical rodent studies, the presence of EEG spikes and their frequency correlate with the later emergence of epilepsy following various brain insults [23,24,27,28,29], including CCI, in adult and immature rodents [23,24]. In vitro studies also show that spikes in hippocampal slices correlate with post-CCI seizure emergence [30], potentially by enhancing excitatory synaptic transmission [27] and inducing axonal sprouting [31]. Even though the high and persistent EEG spiking rate following our modified CCI procedure suggests the emergence of permanent epileptogenic brain circuitry, we did not detect seizures. It is likely that the relatively short 3-month period of EEG recording was not sufficient to detect PTE that commonly emerges as late as 6–9 months post-injury [10,23,32,33,34], echoing the similar long latency period between the injury time and the onset of PTE in humans [35,36].

In our study, the potentiation of hemorrhage in our novel modified CCI procedure induced an increase in hippocampal GFAP levels—a hallmark of reactive astrogliosis that is well described in epileptic foci in both humans and experimental animals [37,38] and may play a crucial role in seizure generation [19,20]. This hippocampal astrogliosis was more prominent on the right side and was exclusively detected in the HTBI rats, paralleling the high frequency of spikes in that group. Even though we did not accurately localize the source of the detected spikes with hippocampal depth electrodes, the co-occurrence of a high frequency of right-predominant spikes with a prominent right-sided hippocampal astrogliosis at the impact site in the HTBI group potentially traces back the origin of the detected spikes to the right hippocampus and suggests that our modified hemorrhage-promoting procedure induced the formation of abnormal hyperexcitable circuitry in the hippocampus. We did not detect an increase in cortical GFAP levels likely due to the higher epileptogenicity of the hippocampus, compared to the cortex, especially in immature rodents [39], and particularly following brain injury in both humans and experimental animals [40,41]. Of note, even though all rats subjected to CCI in our study developed hippocampal damage, only rats subjected to the hemorrhage-promoting modified CCI procedure also developed astrogliosis along with the hippocampal neuronal loss, in a striking similarity to hippocampal sclerosis implicated in seizure emergence in clinical and experimental temporal lobe epilepsy [41,42]. 

Our hemorrhage-potentiating procedure did not worsen the trauma-induced learning deficits in the MWM and the MAAV tests. In line with prior literature, CCI induced deficits in visuospatial navigation in the MWM [43,44,45]. Similarly, both TBI and HTBI groups exhibited deficits in the retention of contextual learning in the MAAV test. As there were no trauma-related motor and sensory deficits, the findings in the MWM and MAAV were attributed to cognitive deficits. The lack of sensory deficits despite the parietal cortical site of impact may be due to the lack or the minimal cortical neuronal loss post-CCI given it was a closed-head injury. As opposed to the TBI group that did not have a cortical neuronal loss, the HTBI group had a statistically significant yet minimal neuronal loss. However, both groups had substantial decreases in hippocampal neuronal densities, which may explain the predominantly hippocampal-related behavioral deficits.

To the best of our knowledge, this is the first study that models hemorrhagic TBI utilizing a pre-CCI heparin treatment as a novel experimental paradigm to potentially better mimic PTE following closed-head injuries in adolescent rats. As rats are much more resistant to bleeding than humans, we used heparin to promote hemorrhage and successfully mimicked a clinical scenario highly associated with PTE. Indeed, our modified CCI procedure consistently and reliably potentiated impact-associated hemorrhage without mortalities, and produced potentially permanent abnormal networks of hyperexcitability in all experimental rats, as evidenced by hippocampal astrogliosis and high EEG spiking rates; essential electrophysiological and pathological landmarks of epileptogenic mechanisms. Even though heparin injections may seem “artificial” and weaken the construct validity of the model, similar injections are commonly needed to better reproduce the electrophysiological and pathological features (face validity) of human epilepsies. Indeed, the most commonly studied rat model of temporal lobe epilepsy is induced with chemoconvulsant injections [42,46,47,48,49,50]. One limitation of our study is that we obtained EEG recordings for only 3 months, and we may have quantified epileptiform discharges during a latency period prior to the emergence of PTE. Our promising results may pave the way for establishing a periadolescent rat model with high rates of PTE and will prompt us to conduct further investigations and extend the EEG recording period for more than 6 months post-injury to detect the possible emergence of PTE.

## 4. Materials and Methods

### 4.1. Animals and Experimental Design

All experiments were approved by the Institutional Animal Care and Use Committee (IACUC) at the American University of Beirut (protocol code 18-02-462, February 2018). Male Sprague Dawley rats were housed in a temperature-controlled room and maintained on a 12 h light–dark cycle with ad libitum access to food and water. At postnatal day 35 (P35), rats were subjected to a single-hit closed-head injury according to the CCI model proposed by Shitaka et al. [51]. Rats were divided into four different groups. The HTBI group (*n* = 16), received a single (600 IU/kg) heparin injection intraperitoneally (i.*p*.) 20 min before the impact, while the TBI group (*n* = 15) received a volume-matched injection of normal saline 20 min pre-impact. HCtrl (*n* = 16), and Ctrl (*n* = 12) groups were subjected to a sham procedure following heparin or normal saline injections, respectively. The administered dose of heparin was determined based on heparin’s known dose-dependent effect on clotting time in rats [52]. A dose of 600 IU/kg was selected to prolong clotting time by fourfold [52] and approximate the human coagulation system at the time of the impact [16]. Following CCI, rats underwent epidural electrodes implantation surgery at P40 and continuous long-term EEG monitoring between P50 and P130. The EEG recordings were interrupted from P80 to P100 for behavioral testing. Rats were sacrificed at P130 for neuronal nuclei (NeuN) staining. An additional cohort of rats (*n* = 3–5 per group) was sacrificed 48 h post-CCI for GFAP staining.

### 4.2. Closed-Head Traumatic Brain Injury Induction

Rats were anesthetized using an intramuscular combination of ketamine (60 mg/kg), xylazine (6 mg/kg), and acepromazine (1.25 mg/kg). Once appropriate anesthesia was achieved (lack of response to toe pinching), the rat was placed on a stereotaxic frame and, the head was firmly secured using two ear bars. A sterile ophthalmic ointment was applied to the eyes to prevent drying. Using aseptic techniques, a midline scalp incision was made and the skin overlying the skull was retracted. The controlled stereotaxic impactor device (ImpactOne^TM^ Stereotaxic impactor for CCI, Leica Biosystems Inc., Wetzlar, Germany) was used to deliver the impact. The coordinates of the impact site (−1.8 mm AP, and 3.6 mm R to bregma) were chosen so that it corresponds to the right parietotemporal region according to the Sherwood and Timiras Atlas of the developing rat brain [53]. The impact was induced with a customized 5 mm flat plastic tip affixed to a pneumatic impactor with an impact duration of 1 s, a depth of 1.5 mm, and a speed of 6.5 m/s. An analgesic paracetamol regimen (1 mg/mL in drinking water) was administered for 3 days following the procedure [54,55]. 

### 4.3. Surgical Electrode Implantation and Continuous Long-Term EEG Recording

Stereotaxic epidural electrode implantation surgery was performed at P40, as previously described [54,56]. Following anesthesia with ketamine, xylazine, and acepromazine, as described in Section 4.2, the rat’s head was secured on a stereotaxic frame, and the skull was exposed. Five small holes were then made using a high-speed driller, and five epidural screw electrodes were placed (1.6 mm in diameter and 4.5 mm in length) including left and right frontal electrodes (2 mm AP, and 3 mm L to bregma), left and right parietal electrodes (−5 mm AP, and 3 mm L to the bregma), and one anterior midline reference electrode (6 mm AP to bregma). A ground electrode was placed under the skin. After the surgery, the rats were transferred to customized single-animal EEG cages [56], and the analgesic regimen with paracetamol (1 mg/mL in drinking water) was administered for 3 days [54,55]. Following a 10-day post-surgical resting period, long-term continuous EEG recordings were initiated. Two blinded readers reviewed the entire EEG tracings for seizures, spikes, and polyspikes. For quantification purposes, two hours were randomly sampled from each rat every 24 h, one from the daytime and one from the nighttime. 

### 4.4. Behavioral Panels

A battery of behavioral tests was selected to assess the commonly reported brain trauma-induced deficits in visuospatial navigation (MWM), learning, and memory (MAAV). Given the impact’s location over the sensory area and its adjacency to the motor area, sensorimotor tests were also performed to assess for potential deficits that may affect the swimming abilities in the MWM and the sensory abilities in the MAAV. Behavioral testing always started at 9 a.m.

The MWM (P80-85) was performed in a dark-blue circular plastic pool (Coulbourn Instruments, Allentown, PA, USA), 150 cm in diameter and 80 cm in height, filled with water (25 °C) to a depth of 30 cm, as previously described [42,57,58]. During habituation, rats were allowed to swim freely for 2 min. During spatial acquisition (days 1–5), a submerged “invisible” escape platform was placed 2 cm below the water surface. Rats underwent four daily trials, and if they failed to find the escape platform in two minutes, they were placed on it for 30 s. During the probe trial on day 6, the platform was removed, and rats were allowed to swim freely for two minutes. On day 7, motor and visual functions were assessed by placing a visible platform. Escape latencies and the time spent in each quadrant were measured with an automated video tracking software (SMART Video Tracking 3.0, Panlab, Harvard Apparatus, Holliston, MA, USA).

The MAAV (P88-94) conditioning test was developed in our laboratory to concurrently assess adaptive avoidance of both context-cued and tone-signaled electrical foot-shocks [57,58,59,60]. The MAAV shuttle box (Coulbourn Instruments, Allentown, PA, USA) consists of two equal compartments connected via an opening in the middle of the partition wall. Following habituation, the right chamber was contextually modified with objects and black and white stripes patterns, while the left chamber was kept plain. During the 6 training days, electrical foot shocks (0.5 mA, 15 s duration) were signaled by a 15 s tone in the left chamber, but in the right chamber, they were delivered if the rat had spent more than 10 s in that compartment. Shuttling between chambers aborted an ongoing shock (escape) or prevented an incoming one (avoidance). During contextual retention testing, rats freely roamed in the box for two minutes, without tone or foot-shock delivery. During auditory retention testing, contextual cues were removed, and 30 tone-signaled trials were delivered in both chambers. The shuttle box was cleaned after each rat with odorless detergent and 70% alcohol solution. 

The rotarod test (P86) consisted of a training phase (one minute at a constant speed of 5 rpm), followed by 3 trials of accelerated speed going from 4 rpm to 40 rpm in 300 s, with a 10 min intertrial resting period. In case the rat passively rotated or fell in less than 5 s, the trial was restarted. 

The Von Frey test (P96) was conducted to test the hind paw’s mechanoreceptor sensitivity using two hair monofilaments (Ugo Basile, Italy) of different forces: the 2 g (19.6 mN), and the 15 g (147 mN) that also assesses nociception. Following 15 min habituation in a transparent box on a metal wire mesh floor, the monofilament was applied to the mid-plantar area of both hind paws with a pressure that causes the filament to buckle. Three trials were performed for each of the two filaments, and each trial consisted of five applications, with 5 min intertrial intervals. 

The Hargreaves test (P97) assessed the latency for hind paw withdrawal in response to a thermal stimulus. Following habituation, a 40% intensity infrared light was applied to the plantar surface of both hind paws. Five applications were performed with 10 min intertrial resting periods. A 20 s cutoff time was set to prevent tissue damage.

### 4.5. Histological Analyses

Rats were sacrificed at P130 to assess cortical and hippocampal neuronal loss with NeuN staining on a sample of 3–5 rats from each group. An additional cohort of rats (*n* = 3–5 per group) was sacrificed 48 h post-impact to assess for cortical and hippocampal reactive astrogliosis (GFAP staining). Brains were perfused with 4% paraformaldehyde via the transcardiac route as previously described [42], then embedded in paraffin. Histological studies were performed on a sample of 3–5 rats from each group. For GFAP analyses, four coronal sections (8 μm) per brain were selected by visual inspection so that their structural patterns match the sections located 2.4–2.7 mm posterior to the bregma according to the Sherwood and Timiras Atlas of the developing rat brain [53]. For NeuN analyses, six coronal sections (8 μm) per brain were selected from the area located 3.3–3.8 mm posterior to the bregma based on the Paxinos and Watson adult rat brain atlas [61]. Immunohistochemistry with 3,3′-Diaminobenzidine (DAB) and hematoxylin counterstaining (Novolink, RE7150-K, Leica Biosystems, Wetzlar, Germany) was performed with anti-GFAP (EnCor Biotechnology, Gainesville, FL, USA) or anti-NeuN (Sigma-Aldrich, Hamburg, Germany) primary antibodies. ImageJ (NIH, Bethesda, MD, USA) was used to measure GFAP optical density and the surface area of the cortical and hippocampal zones selected for NeuN-positive cells manual counting. The parietal cortical zone consisted of a virtual rectangle anchored at the cortical surface. The borders of the hippocampal zone were outlined along the ventricles ventromedially, and the corpus callosum dorsolaterally.

### 4.6. Statistical Analysis

Statistical analyses were performed using Prism 8 (GraphPad Software, San Diego, CA, USA). One-way ANOVA with post hoc Fisher’s least significance difference (LSD) was used to analyze the MWM probe trial and visible platform tests, the MAAV retention subtests, the rotarod test, and the histological data. The EEG and the sensory testing data were analyzed using a two-way ANOVA with post hoc Fisher’s LSD. Two-way repeated-measures ANOVA with post hoc Fisher’s LSD was employed to analyze the MWM and MAAV acquisition phases. Results are reported as means ± S.E.M. (standard error of the mean).

## Data Availability

Not applicable.

## Supplementary Materials

The following are available online at https://www.mdpi.com/article/10.3390/ijms22126456/s1.
