# Supplementary material for: Potentiating Hemorrhage in a Periadolescent Rat Model of Closed-Head Traumatic Brain Injury Worsens Hyperexcitability but Not Behavioral Deficits
Source: Int J Mol Sci. 2021 Jun 16;22(12):6456. doi: 10.3390/ijms22126456 (PMC8234967; doi:10.3390/ijms22126456)
Supplement: Supplementary file 1 [file ijms-22-06456-s001.zip › ijms-1254626-supplementary.pdf]

Supplementary

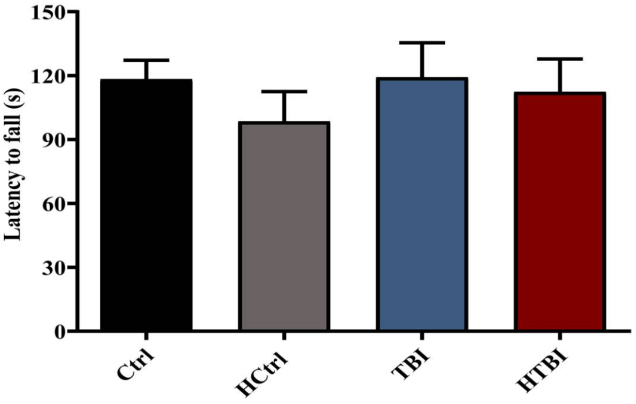

Supplementary Figure S1. Rotarod motor testing. All groups had comparable latencies to fall from the accelerating rotating rod (p>0.05) as revealed by one-way ANOVA with post hoc Fisher's least significance difference (LSD), indicating comparable motor capabilities. (Ctrl: control, n=12; HCtrl: heparinized control, n=16; TBI: traumatic brain injury post-saline injection, n=15; HTBI: traumatic brain injury post-heparin injection, n=16).

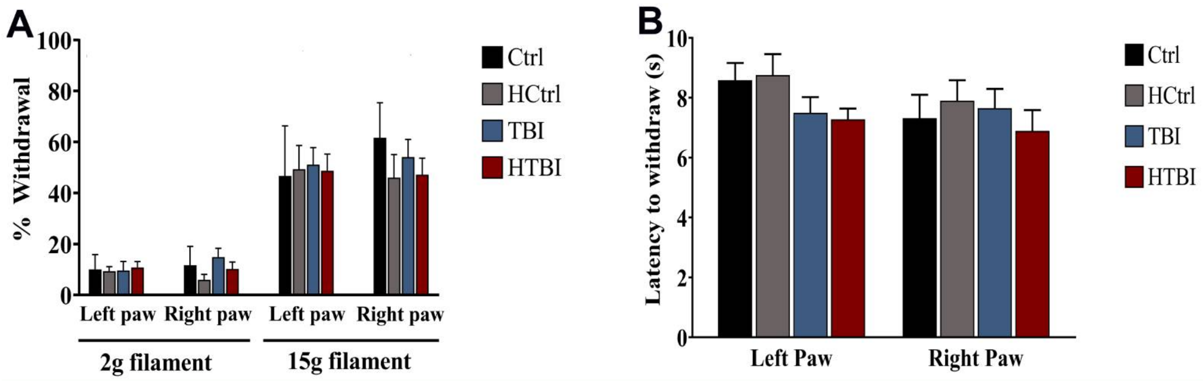

Supplementary Figure S2. Sensory testing. **A.** Shown is the paw withdrawal response to the 2g and 15g filament mechanical stimuli in the Von Frey test. All groups had comparable withdrawal responses in both hind paws (p>0.05) as revealed by two-way ANOVA with post hoc Fisher's least significant difference (LSD). **B:** Shown is the paw withdrawal response to a thermal stimulus in the Hargreaves test. All groups had comparable withdrawal latency of both hind paws (p>0.05, two-way ANOVA followed by post hoc LSD test). (Ctrl: non-heparinized control, n=12; HCtrl: heparinized control, n=16; TBI: traumatic brain injury post-saline injection, n=15; HTBI: traumatic brain injury post-heparin injection, n=16).
